# Leukemia disease burden and quality of care among adolescents and young adults in China compared to global patterns: a comprehensive analysis from 1990 to 2021

**DOI:** 10.3389/fpubh.2026.1772356

**Published:** 2026-05-05

**Authors:** Yi Liu, Menglan Zhu, Lu Gao, Lili Wang

**Affiliations:** 1 Department of Hematology, The 960th Hospital of the PLA Joint Logistics Support Force, Jinan, China; 2 Department of Ophthalmology and Otolaryngology, Unit 32265 of the People’s Liberation Army, Guangzhou, China; 3 Department of Spine Surgery, Shanghai Changzheng Hospital, Shanghai, China; 4 Department of Orthopedics, The 960th Hospital of the PLA Joint Logistics Support Force, Jinan, China

**Keywords:** adolescents and young adults, Bayesian age-period-cohort model, China, disease burden, global burden of disease, leukemia, quality of care index

## Abstract

**Background:**

Leukemia is a major global public health challenge, particularly among adolescents and young adults (AYAs) aged 15–39 years, who face distinct biological and social vulnerabilities. This study aimed to systematically assess long-term trends in leukemia burden and quality of care among Chinese AYAs from 1990 to 2021, and to project future trajectories to inform targeted prevention and control strategies.

**Methods:**

Data were obtained from the Global Burden of Disease (GBD) 2021 database. Temporal trends in incidence, prevalence, years of life lost (YLLs), and years lived with disability (YLDs) among AYAs were analyzed across 204 countries and territories. A Bayesian age–period–cohort (BAPC) model was applied to project incidence and prevalence from 2022 to 2036. A Quality of Care Index (QCI), constructed using principal component analysis, was used to evaluate healthcare performance across different Socio-demographic Index (SDI) regions.

**Results:**

In 2021, the age-standardized incidence rate (ASIR), prevalence rate (ASPR), YLL rate (ASYLLR), and YLD rate (ASYLDR) for leukemia among Chinese AYAs were 3.64, 17.41, 133.55, and 1.87 per 100,000, respectively. The ASYLLR remained moderately elevated compared to high-income Asia-Pacific nations. From 1990 to 2021, China showed a distinct divergent pattern, with ASPR increasing by 145.9% and ASYLDR by 33.6%, while ASYLLR declined by 52.4%. Males consistently experienced a higher burden than females (incidence sex ratio: 1.62:1). The highest premature mortality burden occurred in the 20–24 age group, while the fastest incidence growth was observed among those aged 25–29 years. Acute lymphoblastic leukemia accounted for the greatest burden, whereas chronic lymphocytic leukemia showed the fastest growth despite low incidence. Projections indicated a modest decline in ASIR but a substantial 70.6% increase in ASPR between 2022 and 2036. China’s Quality of Care Index (QCI) reached 74.3%, substantially above the global average (55.2%) and comparable to high-middle SDI regions (71.0%). However, multidimensional analysis revealed specific areas needing improvement, particularly in long-term survivorship care.

**Conclusion:**

Leukemia burden among Chinese AYAs is characterized by rising morbidity alongside declining mortality. These findings provide a national-level reference for macro-level policy formulation and underscore the need for strengthened prevention, improved long-term survivorship care, and more integrated health system responses. Future research should focus on subnational analyses to generate locally actionable evidence.

## 1 Introduction

Leukemia is a group of malignant clonal diseases originating from hematopoietic stem or progenitor cells, constituting a major public health challenge globally (1). According to GBD 2021 data, there were approximately 460,000 new leukemia cases and over 320,000 deaths worldwide in 2021, resulting in approximately 10.98 million disability-adjusted life years (DALYs) of health loss (2, 3). Although global age-standardized incidence rates (ASIR) and mortality rates have generally declined over the past 30 years due to medical advances and treatment optimization, significant disparities persist across countries and regions, and absolute case numbers continue to rise due to population growth (4, 5).

AYAs aged 15–39 years represent a particularly important yet relatively neglected group in leukemia burden research (6). This age group is at critical stages of education, employment, and family establishment, representing the core workforce of society (7). Leukemia not only imposes substantial physical and psychological burden on patients but also profoundly affects family economics and social productivity (8). Compared with pediatric and older patients, young adult leukemia patients exhibit unique disease spectrum characteristics, treatment responses, and long-term prognoses: ALL incidence in young adults is significantly lower than in children, while the relative proportion of chronic leukemias gradually increases. Additionally, young patients face special challenges including fertility preservation, career interruption, and psychosocial adaptation, which have received insufficient attention in existing research.

As the world’s most populous country, China presents complex epidemiological characteristics of leukemia burden. Recent studies indicate that Chinese leukemia incidence has shown fluctuating upward trends over the past three decades, with an annual growth rate of 1.9% during 2005–2017 (9, 10). Notably, leukemia burden among Chinese AYAs is significantly higher than in developed countries such as the United States (11).

Multiple environmental and behavioral risk factors may contribute to this elevated burden, warranting systematic investigation (12, 13). Although China has made significant progress in leukemia diagnosis and treatment in recent years—with childhood ALL 5-year overall survival rates improving from less than 65% before 2014 to approximately 90% after 2015 (14, 15)—systematic analysis of nationwide disease burden evolutionary trends, regional variations, and quality of care assessment remains lacking. Existing studies mostly focus on specific leukemia subtypes or regional data, lacking comprehensive burden assessment for young adult populations based on nationally representative data. Furthermore, while the association between SDI and leukemia burden has been widely recognized (16), systematic evaluation indicator systems for young adult care quality have not been established, making it difficult to objectively reflect quality gaps in disease prevention, diagnosis, treatment, and rehabilitation across countries and regions with different development levels.

More importantly, scientific projection of future leukemia burden is of great significance for health policy formulation and medical resource allocation in the context of population aging and disease spectrum transition (17). The BAPC model, by integrating age, period, and cohort effects, can more accurately capture complex nonlinear trends in disease burden and provide probabilistic predictions, demonstrating superior performance over traditional models in burden projection (18). However, medium- to long-term predictions of future leukemia burden among Chinese young adults remain very limited.

Against this background, this study systematically analyzed spatiotemporal distribution characteristics and evolutionary trends of leukemia burden among Chinese AYAs aged 15–39 from 1990 to 2021 based on the GBD 2021 database, and employed BAPC models to project burden trajectories for 2022–2036. Simultaneously, by constructing a QCI, we multi-dimensionally evaluated gaps in leukemia care quality between China and different SDI regions globally. This study aims to provide scientific evidence for optimizing Chinese AYA leukemia prevention and control strategies, improving care quality, and rationally allocating medical resources, while supporting data for achieving the “Healthy China 2030” Planning Outline goal of reducing premature mortality from major chronic diseases.

## 2 Materials and methods

### 2.1 Data sources

#### 2.1.1 Data source and study population

This study used the GBD 2021 database as the data source, including leukemia-related data for populations aged 15–39 years across 204 countries and territories globally, as well as China. Leukemia cases were defined according to ICD-10 codes C91-C95, including five subtypes: acute lymphoblastic leukemia (ALL), chronic lymphocytic leukemia (CLL), acute myeloid leukemia (AML), chronic myeloid leukemia (CML), and other leukemias (1). The study population was stratified by 5-year age groups (15–19, 20–24, 25–29, 30–34, and 35–39 years), with population data from World Population Prospects 2022 and standardization using the GBD 2019 global standard population (19). Countries and territories were classified into five SDI levels: low, low-middle, middle, high-middle, and high SDI.

To address potential limitations of secondary analysis, this study controlled for information bias, selection bias, and ecological fallacy through standardized diagnostic processing, 95% uncertainty interval (UI) assessment, and multi-model validation. Missing data were imputed using spatiotemporal Gaussian process regression (ST-GPR) (20).

#### 2.1.2 Geographic scope and granularity

This study analyzes national-level aggregate data for China. We acknowledge this as a significant limitation, as China exhibits substantial geographic heterogeneity in socioeconomic development (provincial SDI ranges from ~0.52 to ~0.89), healthcare infrastructure, and environmental exposures. The GBD 2021 database does not provide provincial-level estimates for China, and China’s National Cancer Registry has incomplete geographic coverage (~38% of national population as of 2018) with variable data quality. National aggregate findings may therefore obscure within-country heterogeneity and limit actionability for provincial health departments. Consequently, the national-level findings presented here should be interpreted as providing a broad overview of national trends and international context. This aggregate perspective may obscure significant within-country heterogeneity, potentially leading to an ecological fallacy where national averages do not accurately reflect the situation in any specific province. Therefore, these results are best viewed as hypothesis-generating for more granular, subnational analyses, rather than as precise guidance for provincial health departments.

### 2.2 Study indicators

Primary study indicators included ASIR, age-standardized prevalence rate (ASPR), age-standardized years of life lost rate (ASYLLR), and age-standardized years lived with disability rate (ASYLDR). DALYs equal the sum of years of life lost (YLLs) and years lived with disability (YLDs) (21). Absolute case numbers (incident cases, prevalent cases) were extracted to complement rate-based measures for assessing absolute disease burden.

The estimated annual percentage change (EAPC) was used to assess temporal trends in disease burden from 1990 to 2021. EAPC was calculated as: EAPC = 100 × (exp(*β*) − 1), where β is the regression coefficient of ln(age-standardized rate) on calendar year. EAPC and its 95% confidence interval (CI) were obtained through linear regression models. A trend was considered increasing when both EAPC and its 95% CI lower limit were > 0, decreasing when both EAPC and its 95% CI upper limit were < 0, and stable otherwise (22). The cumulative percentage change over the 30-year period was calculated as (exp(*β* × 30) − 1) × 100% to quantify long-term changes.

### 2.3 Statistical analysis

#### 2.3.1 Disease burden trend projection

To project leukemia incidence and prevalence trends from 2022 to 2036, a BAPC model was constructed. The model achieved Bayesian statistical inference through the INLA package in R software, using integrated nested Laplace approximation for parameter estimation (23). The BAPC model expression is:


log(λijk)=α+μi+βj+γk+εijk

Where *λijk* is the expected observation value, *α* is the intercept, *μi* is the age effect, *βj* is the period effect, *γk* is the cohort effect, and *εijk* is the random error term. Age effects used second-order random walk priors, while period and cohort effects used first-order random walk priors.

Model quality was assessed using deviance information criterion (DIC) and Watanabe-Akaike information criterion (WAIC), with smaller values indicating better model fit. Model assumptions were validated through residual normality tests and autocorrelation tests, and prediction validity was evaluated using leave-one-out cross-validation. Prediction results were presented as point estimates with 95% credible intervals.

#### 2.3.2 Quality of care index construction

The QCI was constructed using principal component analysis (PCA), based on four secondary indicators: early diagnosis index (YLL/YLD ratio), burden control index (DALYs/prevalence ratio), treatment standardization index (deaths/incidence ratio), and recovery efficiency index (prevalence/incidence ratio) (24).

First, the four basic indicators were Z-score standardized, followed by PCA to extract principal components. The first two principal components explained 98.59% of the total variance (PC1: 94.27%, PC2: 4.32%), indicating excellent dimensionality reduction. An improved formula combining the first two principal components weighted by variance was used:


QCI_PCA=(PC1×Var1+PC2×Var2)/(Var1+Var2)

Where PC1 and PC2 are the first and second principal component scores, and Var1 and Var2 are the corresponding explained variance proportions. The PCA index was then converted to a 0–100 range through Min–Max standardization:


QCI=100×(QCI_PCA−QCI_min)/(QCI_max−QCI_min)

Higher final QCI values indicate better quality of care.

#### 2.3.3 Correlation analysis

Smooth spline models were used to analyze the nonlinear association between age-standardized DALY rate (ASDR) and SDI, combined with locally weighted scatterplot smoothing (LOWESS) for curve fitting. Spearman rank correlation analysis was used to quantify the strength of correlation between QCI and SDI, calculating the correlation coefficient *ρ* and its statistical significance (two-sided tests, *p* < 0.05 considered statistically significant).

All statistical analyses were performed using R software (version 4.3.0). Statistical significance was determined using a threshold of p < 0.05, with 95% CI (or credible intervals) not containing zero as the criterion for significant differences.

#### 2.3.4 Statistical significance assessment

All statistical analyses were performed using R software (version 4.3.0). All statistical analyses were performed using R software (version 4.3.1). For descriptive analyses and model-based inferences, such as the Estimated Annual Percentage Change (EAPC) and Bayesian projections, statistical significance was determined by whether the 95% confidence intervals (or 95% credible intervals) excluded the null value (e.g., zero for EAPC). For comparative analyses, including the Wilcoxon signed-rank test for sex differences and Spearman rank correlation for associations, a two-sided *p*-value of less than 0.05 was considered statistically significant. All statistical test results are provided in Supplementary Tables S1, S2.

## 3 Results

### 3.1 Overall disease burden

**Figure 1:**
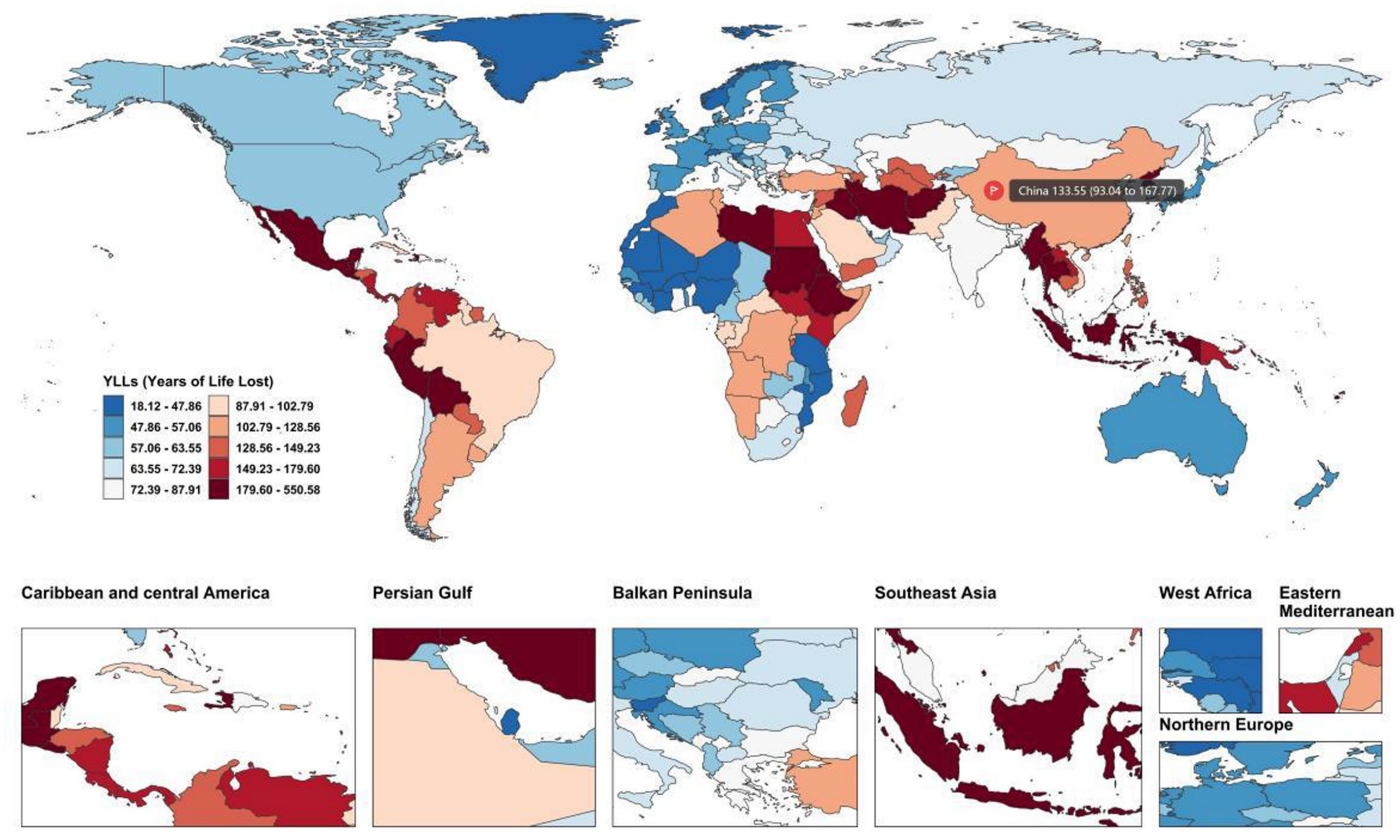
Global distribution of age-standardized years of life lost (YLL) rates for leukemia among adolescents and young adults aged 15–39 years in 2021. The map displays the global distribution of ASYLLR, with darker colors indicating higher disease burden. High-burden regions are concentrated in sub-Saharan Africa, the Middle East, and parts of Latin America.

Globally, leukemia disease burden among the 15–39 age group showed wide geographical disparities (Figure 1). High-burden regions were mainly concentrated in sub-Saharan Africa (Ethiopia: 548.3 per 100,000, 95% UI: 374.5–787.2; Kenya: 154.2, 95% UI: 111.8–205.8), the Middle East (Afghanistan: 284.7, 95% UI: 166.1–417.6), and parts of Latin America (Bolivia: 270.0, 95% UI: 206.8–349.1), where YLL rates were 1.7–6.0 times higher than the global average (91.82 per 100,000, 95% UI: 73.61–103.34).

The ASYLLR for leukemia among Chinese AYAs aged 15–39 was 133.55/100,000 (95% UI: 93.04–167.77). This value was lower than high-burden regions (such as Ethiopia and Afghanistan with ASYLLR exceeding 250/100,000) but higher than developed countries (Japan: 48.40/100,000; Australia: 49.44/100,000; most Western European countries: 40–60/100,000). Compared with East Asian countries, China’s ASYLLR was higher than Japan but lower than most Southeast Asian countries (Thailand: 193.42/100,000, 95% UI: 150.73–257.38; Indonesia: 189.76/100,000, 95% UI: 137.30–255.15; Philippines: 138.32/100,000). China’s ASYLLR was approximately 1.5-fold higher than the global average (91.82, 95% UI: 73.61–103.34).

In 2021, the population-wide ASYLLR in China was 133.55/100,000, far exceeding ASIR (3.64/100,000) and ASYLDR (1.87/100,000). Males showed higher burden across all indicators (incidence ratio 1.62:1, *p* < 0.0001).

Leukemia subtypes showed distinct burden profiles (Table 1). ALL had the highest age-standardized burden (ASPR 6.61/100,000; ASYLDR 0.79/100,000; YLLs 243,844 cases). CLL had the lowest ASIR (0.55/100,000) but the highest absolute prevalent cases (61,396, 48.6% of total) and incident cases (7,165, 32.6% of total), surpassing ALL (prevalent cases 29,570, 23.4%; incident cases 6,644, 30.2%). CLL showed the fastest growth (ASIR EAPC 1.963%; ASYLDR EAPC 2.924%). CML showed declines (ASIR EAPC -2.093%; ASYLLR EAPC -5.188%).

**Table 1:** Age-standardized rates of leukemia among Chinese youth aged 15–39 years in 2021 and their estimated annual percentage changes (EAPC) from 1990 to 2021.

Variable	Subgroup	ASIR		ASPR		ASYLLR		ASYLDR	
		Rate, 2021	EAPC, 1990–2021	Rate, 2021	EAPC, 1990–2021	Rate, 2021	EAPC, 1990–2021	Rate, 2021	EAPC, 1990–2021
Overall		3.64 (2.47 to 4.58)	−0.126 (−0.245 to −0.008)	17.41 (11.27 to 22.28)	1.978 (1.849 to 2.107)	133.55 (93.04 to 167.77)	−1.681 (−1.805 to −1.557)	1.87 (1.12 to 2.75)	1.01 (0.876 to 1.144)
Sex	Female	2.74 (1.56 to 3.69)	−0.766 (−0.846 to −0.685)	13.04 (6.81 to 18.09)	1.219 (1.138 to 1.299)	101.19 (61.63 to 135.29)	−2.223 (−2.349 to −2.096)	1.36 (0.71 to 2.06)	0.257 (0.177 to 0.338)
	Male	4.45 (2.57 to 6.01)	0.307 (0.156 to 0.459)	21.36 (11.69 to 29.3)	2.504 (2.316 to 2.692)	162.70 (96.37 to 218.51)	−1.322 (−1.459 to −1.185)	2.32 (1.20 to 3.54)	1.505 (1.331 to 1.679)
Age (years)	15–19	3.49 (2.28 to 4.48)	−0.794 (−0.986 to −0.602)	15.02 (9.17 to 19.69)	2.754 (2.358 to 3.151)	144.11 (98.88 to 183.02)	−2.594 (−2.763 to −2.425)	1.62 (0.94 to 2.37)	0.793 (0.491 to 1.097)
	20–24	3.73 (2.49 to 4.69)	0.374 (0.226 to 0.523)	18.69 (11.71 to 24.14)	2.562 (2.405 to 2.720)	151.21 (104.23 to 188.94)	−1.323 (−1.469 to −1.176)	2.08 (1.22 to 3.12)	1.528 (1.361 to 1.695)
	25–29	3.43 (2.28 to 4.27)	0.690 (0.516 to 0.864)	17.65 (11.23 to 21.99)	2.589 (2.392 to 2.786)	121.91 (83.76 to 152.45)	−0.896 (−1.036 to −0.755)	1.80 (1.04 to 2.66)	1.774 (1.569 to 1.979)
	30–34	3.47 (2.42 to 4.33)	0.043 (−0.082 to 0.169)	16.34 (11.14 to 20.87)	1.592 (1.446 to 1.737)	121.62 (86.41 to 151.57)	−1.227 (−1.350 to −1.103)	1.77 (1.07 to 2.58)	0.895 (0.768 to 1.022)
	35–39	4.13 (2.92 to 5.19)	−0.681 (−0.840 to −0.522)	19.74 (13.51 to 25.15)	0.796 (0.655 to 0.937)	126.32 (90.62 to 159.46)	−1.902 (−2.088 to −1.715)	2.08 (1.35 to 3.05)	0.182 (0.041 to 0.324)
Type	Acute lymphoid leukemia	1.65 (0.86 to 2.17)	−0.361 (−0.603 to −0.119)	6.61 (3.38 to 8.84)	3.055 (2.508 to 3.606)	65.25 (35.20 to 85.50)	−1.858 (−1.972 to −1.744)	0.79 (0.37 to 1.20)	1.165 (0.764 to 1.568)
	Chronic lymphoid leukemia	0.55 (0.30 to 0.79)	1.963 (1.783 to 2.143)	4.16 (2.24 to 6.05)	3.414 (3.116 to 3.712)	13.34 (7.32 to 18.99)	−0.572 (−0.755 to −0.389)	0.39 (0.20 to 0.63)	2.924 (2.670 to 3.178)
	Acute myeloid leukemia	0.68 (0.42 to 0.95)	−0.519 (−0.728 to −0.309)	1.69 (1.07 to 2.37)	0.124 (−0.128 to 0.376)	32.69 (20.88 to 46.96)	−1.223 (−1.471 to −0.973)	0.22 (0.13 to 0.32)	−0.291 (−0.511 to −0.072)
	Chronic myeloid leukemia	0.17 (0.08 to 0.26)	−2.093 (−2.419 to −1.765)	0.43 (0.19 to 0.67)	1.120 (0.850 to 1.389)	3.71 (1.84 to 6.12)	−5.188 (−5.605 to −4.771)	0.05 (0.02 to 0.09)	−1.268 (−1.583 to −0.951)
	Other leukemia	0.60 (0.27 to 0.84)	0.132 (0.030 to 0.235)	4.52 (2.04 to 6.30)	0.798 (0.687 to 0.910)	21.49 (9.86 to 30.05)	−1.258 (−1.371 to −1.145)	0.41 (0.17 to 0.66)	0.464 (0.359 to 0.570)

ASIR, Age-Standardized Incidence Rate; ASPR, Age-Standardized Prevalence Rate; ASYLLR, Age-Standardized Years of Life Lost Rate; ASYLDR, Age-Standardized Years Lived with Disability Rate; EAPC, Estimated Annual Percentage Change; ALL, Acute Lymphoblastic Leukemia; CLL, Chronic Lymphocytic Leukemia; AML, Acute Myeloid Leukemia; CML, Chronic Myeloid Leukemia.

All values are presented as the estimate (95% uncertainty interval). Rates are expressed per 100,000 population. EAPC values represent the trend over the period 1990 to 2021.

### 3.2 Comparison of leukemia disease burden between China and other regions

Two-way ANOVA showed significant main effects of region and year for all burden indicators (all *p* < 0.001, Table 2) and significant region × year interactions (*F* = 65.88 to 263.45, all *p* < 0.001, η^2^ = 0.011 to 0.057).

**Table 2:** Two-way ANOVA results for leukemia disease burden indicators across regions and time, 1990–2021.

Indicator	Location effect			Year effect			Location × Year interaction		
	*F*(6,210)	*p*	η^2^	*F*(1,210)	*p*	η^2^	F(6,210)	*p*	η^2^
ASIR	5696.06	<0.001	0.943	1446.30	<0.001	0.040	65.88	<0.001	0.011
ASPR	4239.30	<0.001	0.910	711.37	<0.001	0.025	263.45	<0.001	0.057
ASYLDR	5125.61	<0.001	0.963	15.46	<0.001	0.000	157.73	<0.001	0.030
ASYR	3662.73	<0.001	0.747	5898.72	<0.001	0.201	220.70	<0.001	0.045

Results from two-way analysis of variance (ANOVA) with region as a categorical variable and year as a continuous variable. ASIR, age-standardized incidence rate; ASPR, age-standardized prevalence rate; ASYLDR, age-standardized years lived with disability rate; ASYR, age-standardized years of life lost rate. F, F-statistic; df, degrees of freedom (numerator, denominator); η^2^, eta-squared (effect size, proportion of variance explained). All disease burden rates are per 100,000 population.

**Figure 2:**
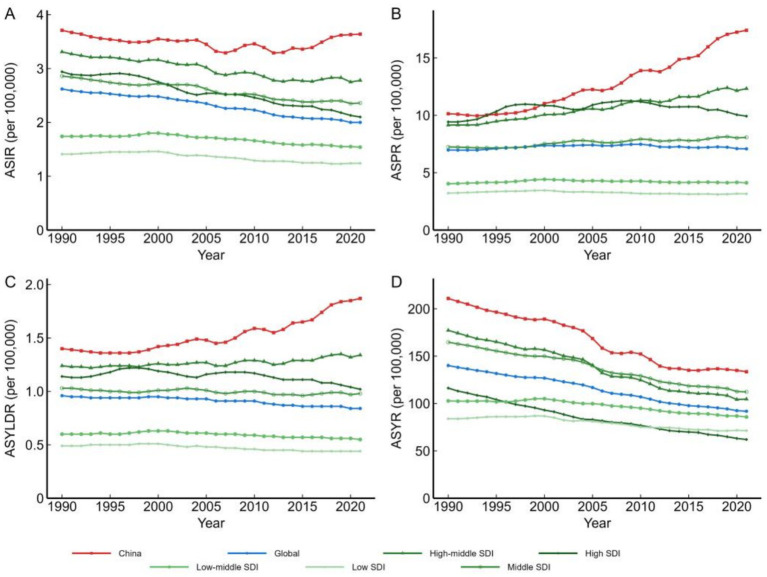
Temporal trends in age-standardized disease burden indicators for leukemia among youth aged 15–39 years in China compared with other regions, 1990–2021. Line charts showing trends in ASIR **(A)**, ASPR **(B)**, ASYLLR **(C)**, and ASYLDR **(D)** across different SDI regions and China.

In 2021, China’s ASPR reached 17.41/100,000, a 145.9% increase from 1990. China’s ASYLDR increased by 33.6%, while the global ASYLDR declined by 12.5% (Figure 2). China’s ASIR declined by 1.9%, compared with the global decline of 23.7%. As a high-middle SDI region, China’s ASPR and ASYLDR trends differed from other regions in the same group (all pairwise comparisons *p* < 0.001).

### 3.3 Age and sex distribution characteristics

**Figure 3:**
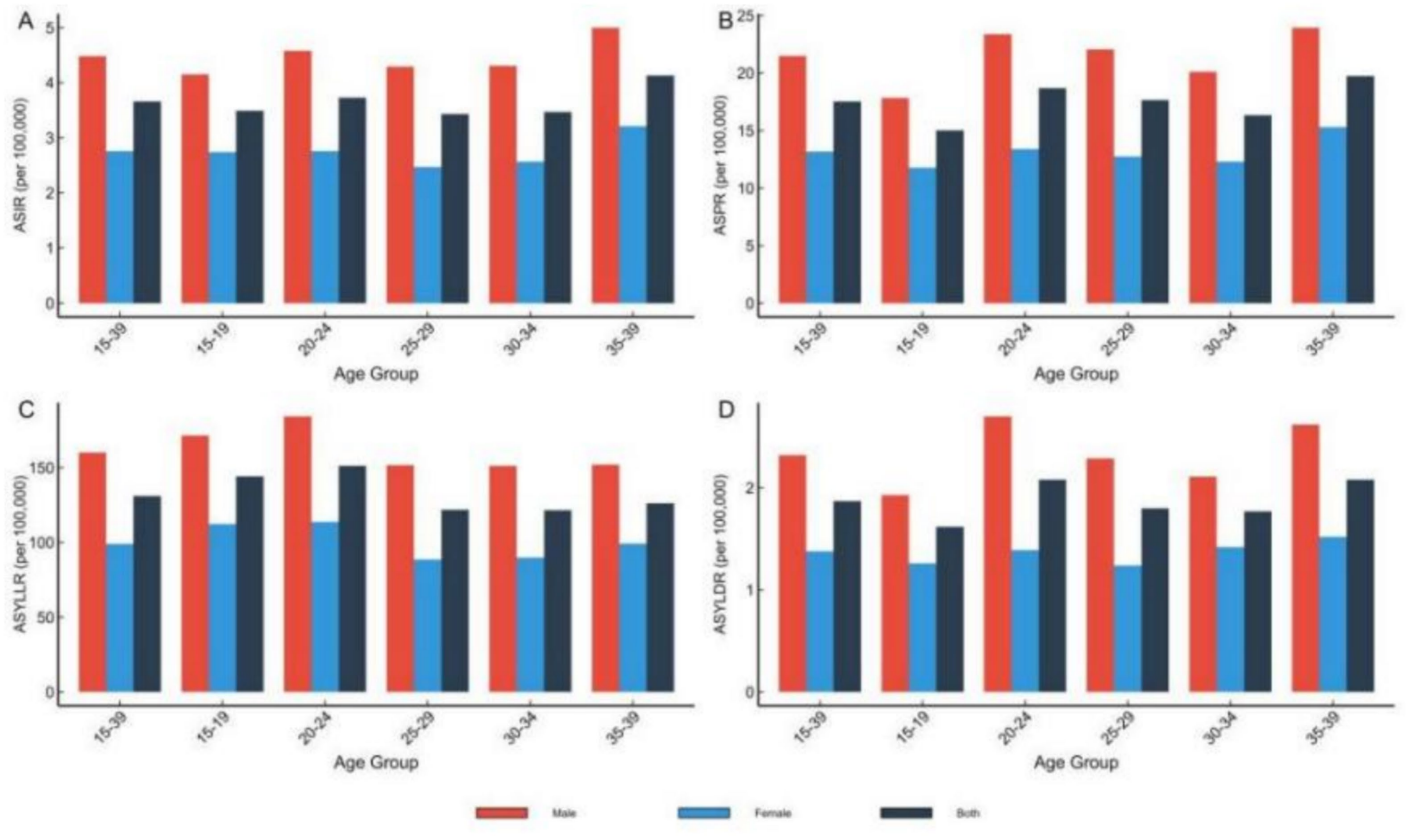
Disease burden of leukemia among Chinese youth aged 15–39 years by age group, 2021. Bar charts displaying ASIR **(A)**, ASPR **(B)**, ASYLLR **(C)**, and ASYLDR **(D)** stratified by 5-year age groups and sex.

In 2021, the 35–39 age group had the highest ASIR (4.13/100,000), while the 20–24 age group had the highest ASPR (18.69/100,000). Male burden was higher than female across all age groups (*p* < 0.0001, Wilcoxon signed-rank test), with male-to-female incidence ratios 1.56–1.74 and prevalence ratios 1.57–1.75 (all *p* < 0.0001) (Figure 3).

**Figure 4:**
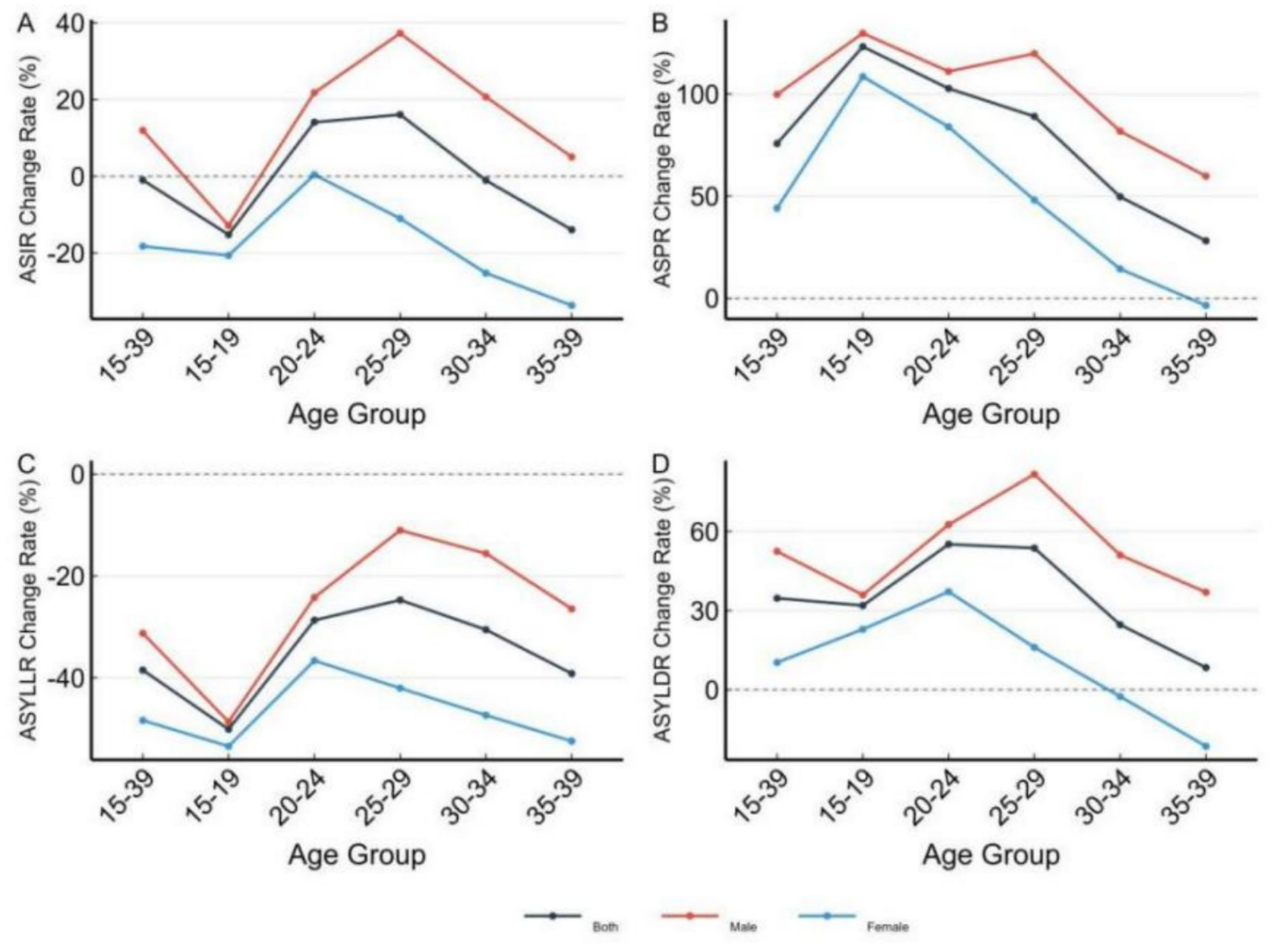
Percentage change in disease burden of leukemia among Chinese youth aged 15–39 years by age group, 1990–2021. Heatmap showing the percent change in ASIR **(A)**, ASPR **(B)**, ASYLLR **(C)**, and ASYLDR **(D)** across age groups and sex categories.

From 1990 to 2021, overall ASIR for ages 15–39 remained stable (−0.99%), but increased in the 20–29 group (+14.1% to +16.1%) and decreased in the 15–19 (−15.2%) and 35–39 (−13.9%) groups. ASPR increased across all age groups, most notably in 15–19 (+123.2%, *p* = 0.003), 20–24 (+102.7%, *p* = 0.006), and 25–29 (+89.1%, *p* = 0.007). ASYLLR declined significantly in several groups, including 15–19 (−50.1%, *p* < 0.001) and 35–39 (−39.2%, *p* = 0.009). ASYLDR showed increases in some groups, particularly males aged 20–29 (Figure 4).

### 3.4 Projected leukemia incidence and prevalence trends for 2022–2036

PrASIR fluctuated downward to 3.391/100,000 in 2016, then rebounded to 3.568/100,000 in 2022. Projections showed a decline to 3.335/100,000 (95% CI 2.626–4.043) by 2035.

ASPR increased from 10.13/100,000 in 1990 to 17.42/100,000 in 2021, projected to reach 33.08/100,000 (95% CI 26.04–40.11) by 2035.

**Figure 5:**
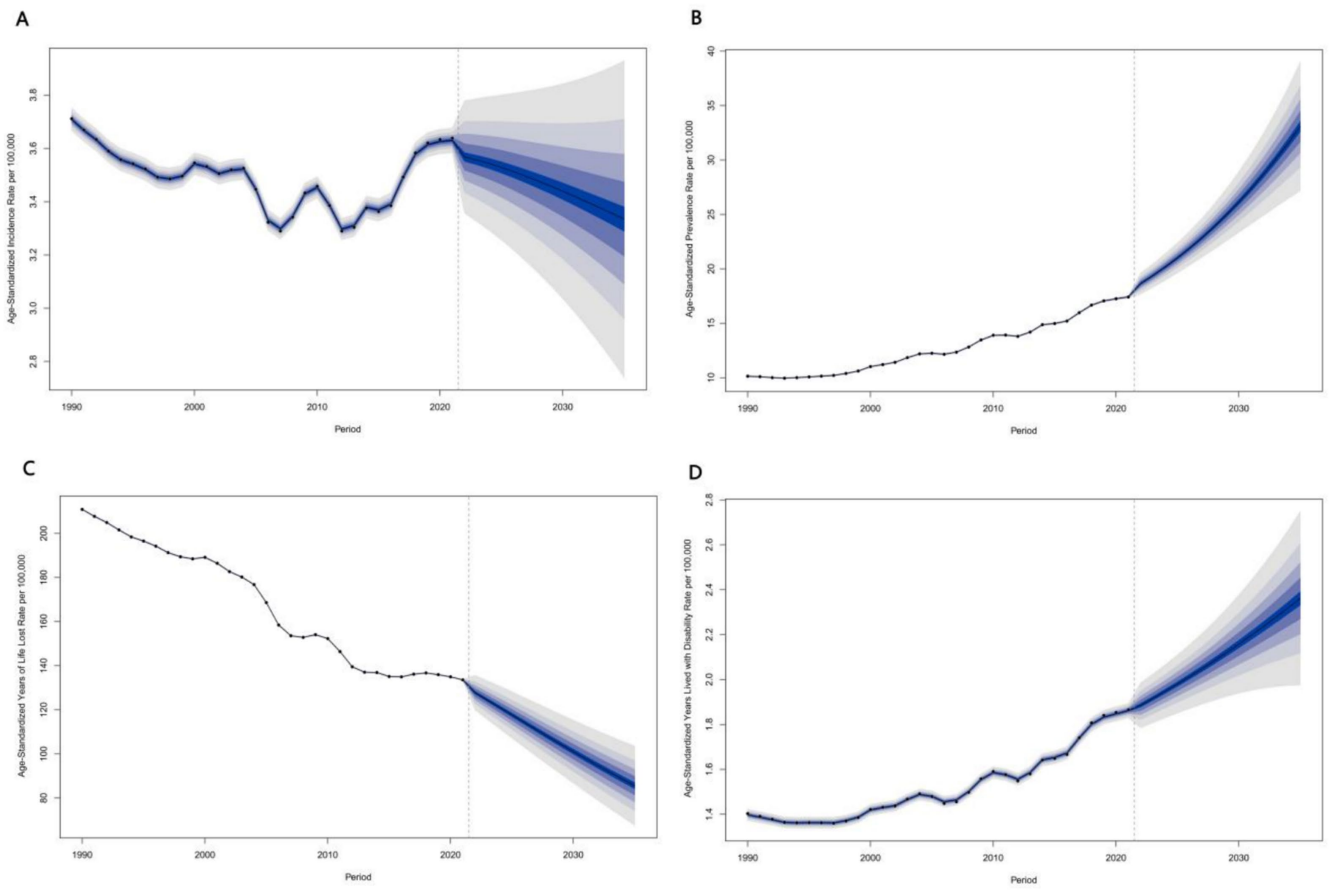
Projected trends in age-standardized incidence **(A)**, prevalence **(B)**, YLL **(C)**, and YLD rates of leukemia among Chinese youth aged 15–39 years, 2022–2036. Projections based on Bayesian age-period-cohort (BAPC) model with 95% credible intervals shown as shaded areas.

ASYLLR declined from 210.89/100,000 in 1990 to 124.43/100,000 in 2023, projected to 85.58/100,000 (95% CI 64.17–106.99) by 2035. ASYLDR rose from 1.40/100,000 in 1990 to 1.86/100,000 in 2021, projected to 2.36/100,000 (95% CI 1.90–2.82) by 2035 (Figure 5). Confidence intervals widened after 2030.

### 3.5 Correlation analysis and quality of care index construction

**Figure 6:**
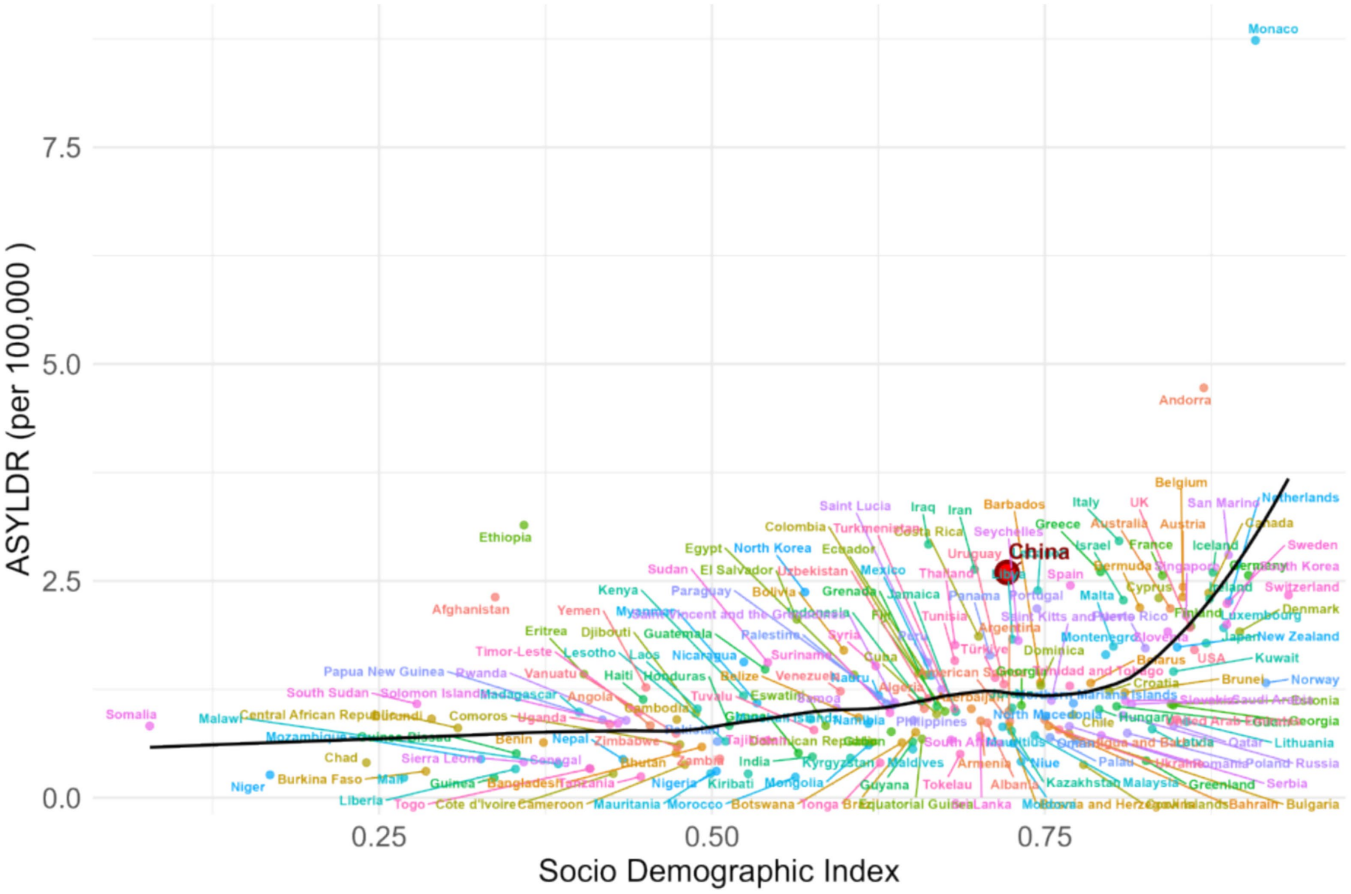
Smooth spline fit analysis of the age-standardized years lived with disability (YLD) rate for leukemia and the Socio-demographic Index (SDI). Scatter plot with fitted curve showing the positive correlation between ASYR (age-standardized YLD rate) and SDI (*ρ* = 0.551, *p* < 0.001).

**Figure 7:**
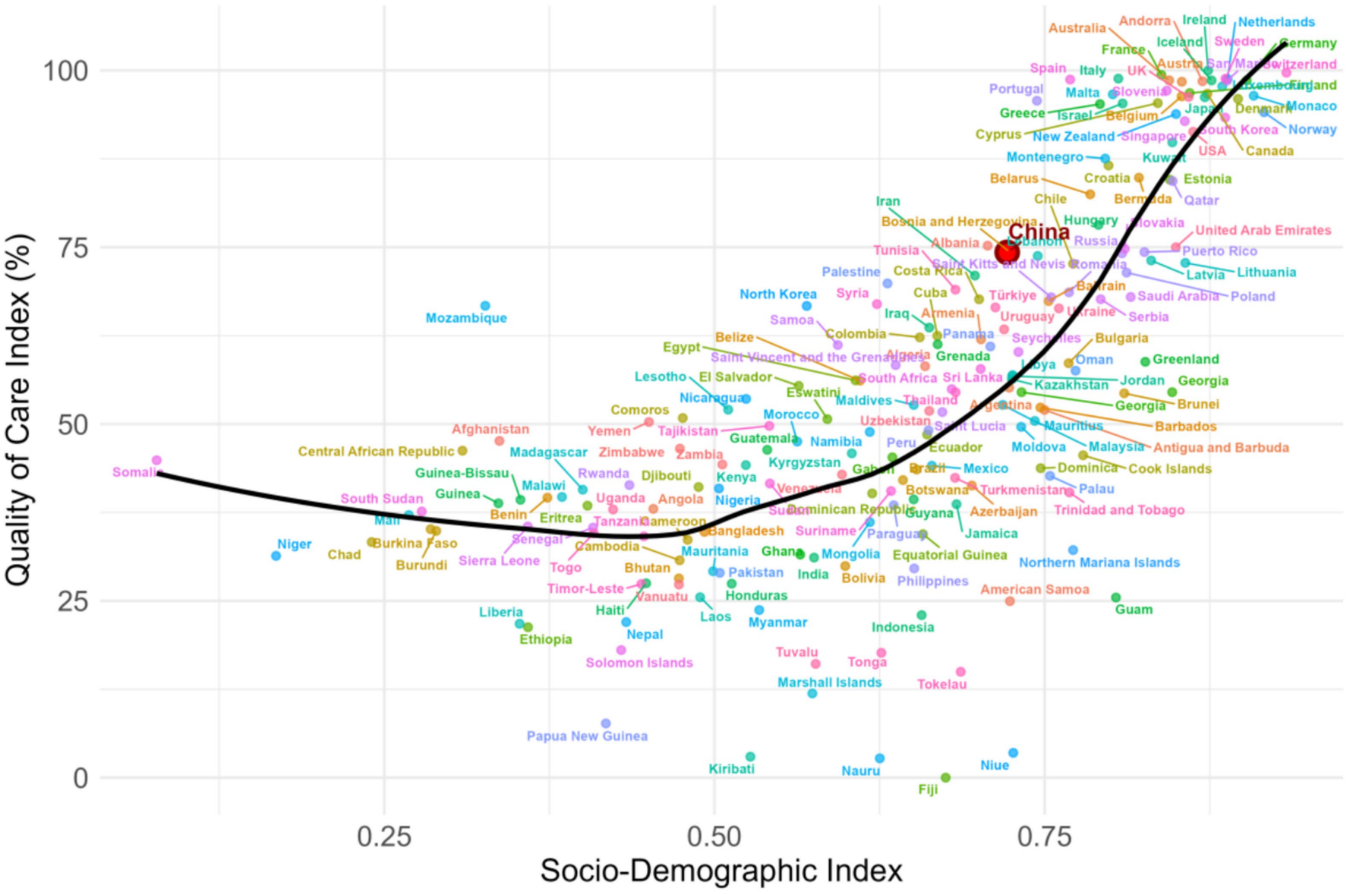
Correlation between quality of care index (QCI) and socio-demographic index (SDI) for leukemia in individuals aged 15–39 years, 2021. Scatter plot demonstrating the strong positive correlation between QCI and SDI (Spearman’s ρ = 0.77, p < 0.001). Each point represents a country or territory. China’s position is highlighted (SDI = 0.722, QCI = 74.3%), showing its QCI is substantially above the global average and comparable to high-middle SDI regions.

**Figure 8:**
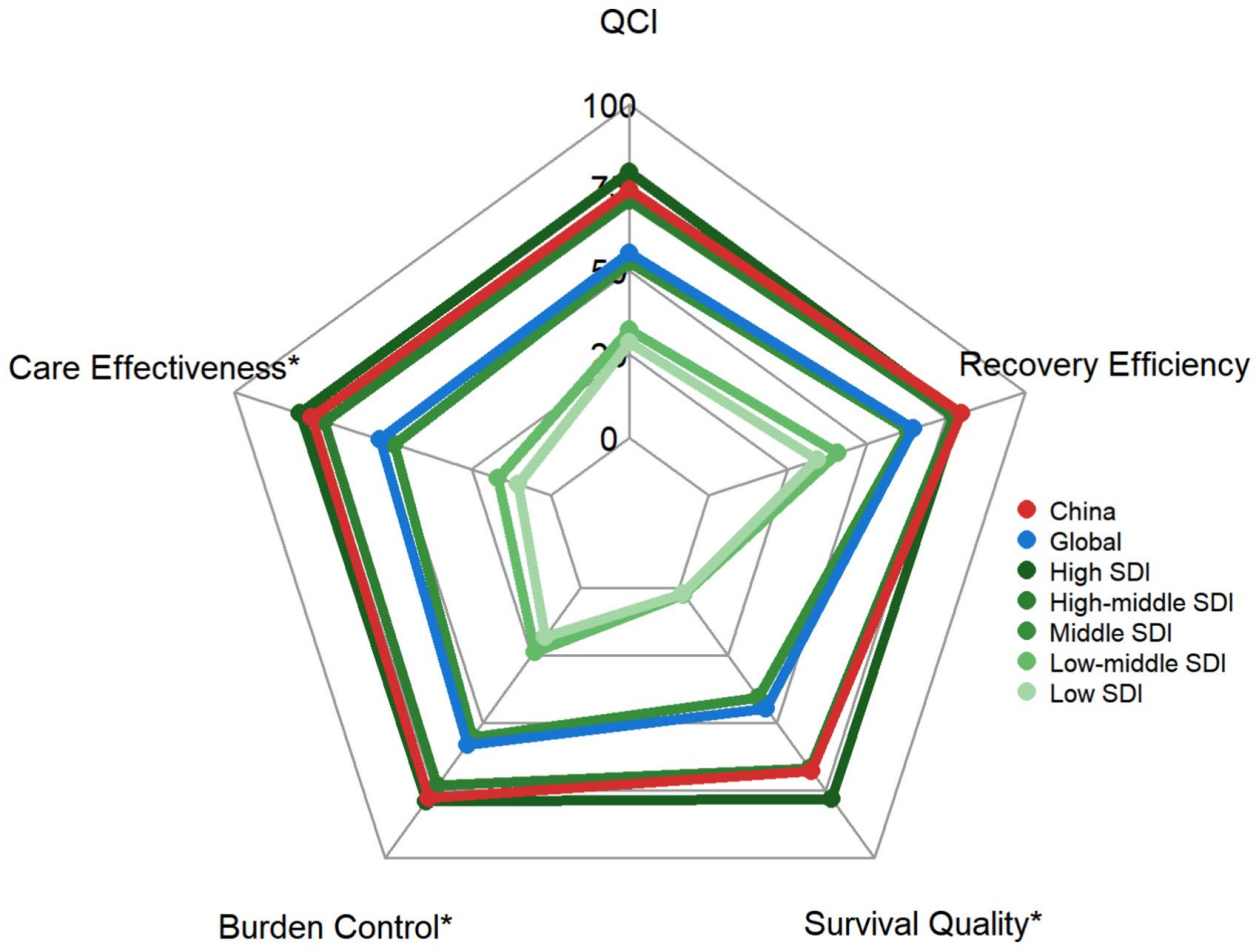
Quality of care index components for leukemia in young adults aged 15–39 years by SDI region, 2021: a radar chart analysis. The five dimensions include care effectiveness index, burden control index, survival quality index, recovery efficiency index, and comprehensive QCI. *Care effectiveness, burden control, and survival quality are inverted from original negative indicators.

Spearman correlation showed a moderate positive association between ASYLDR and SDI (*ρ* = 0.551, *p* < 2.2e-16) (Figure 6). QCI showed a strong positive correlation with SDI (ρ = 0.772, *p* < 2.2 × 10^−16^) (Figure 7). China’s QCI was 74.3% (SDI 0.722), above the global average (55.2%) and comparable to high-middle SDI regions (71.0%). China outperformed global averages in all five dimensions: care effectiveness 75.5% (global 53.9%), burden control 77.8% (global 58.0%), survival quality 67.7% (global 44.7%), recovery efficiency 79.6% (global 64.5%) (Figure 8).

## 4 Discussion

This study systematically analyzed the characteristics of leukemia disease burden among Chinese adolescents and young adults (AYAs) aged 15–39 from 1990 to 2021 using the GBD 2021 database, projected developmental trends for 2022–2036, and constructed a Quality of Care Index (QCI) to assess care levels across different global regions. The results revealed unique epidemiological patterns of leukemia burden among Chinese young adults, providing important scientific evidence for disease prevention, control, and care quality improvement.

### 4.1 Major findings and international comparison

#### 4.1.1 China’s position in the global leukemia burden landscape and interpretation framework

China occupies an intermediate position in the global AYA leukemia burden landscape. Its age-standardized years of life lost rate (ASYLLR) was 133.55 per 100,000, which is 2.6 times higher than that of high-income Asia-Pacific countries (Japan: 48.40; Australia: 49.44), yet substantially lower than high-burden regions (Ethiopia: 548.3; Afghanistan: 282.4) and neighboring Southeast Asian countries (Thailand: 191.7; Indonesia: 188.7). This positioning broadly aligns with China’s high-middle Sociodemographic Index (SDI) status, but the persistent gap with high-income Asian neighbors warrants explanation.

Three structural factors explain this gap. First, delayed implementation of standardized treatment protocols: while Japan integrated multi-institutional collaborative networks and evidence-based guidelines in the 1990s, widespread adoption in China occurred primarily after 2010 (14, 25). Second, unequal geographic distribution of hematology expertise: advanced leukemia care remains concentrated in tier-1 cities, with substantial treatment delays and quality variations in lower-tier cities and rural areas (26). Third, later adoption of cost-effective interventions: for example, tyrosine kinase inhibitors for chronic myeloid leukemia (CML) entered Japan’s healthcare system in 2001 but achieved widespread accessibility in China only after inclusion in medical insurance around 2010 (27, 28).

However, international comparisons require careful interpretation, considering three methodological factors. First, China’s rapid expansion of advanced diagnostic capabilities over the past two decades means early-period data may reflect under-ascertainment of cases rather than truly lower disease occurrence, potentially inflating observed temporal increases. Second, genetic heterogeneity affects baseline risk—East Asian populations exhibit distinct leukemia susceptibility patterns compared to European or African populations, complicating cross-ethnic comparisons. Third, SDI captures only aggregate socioeconomic development and does not measure healthcare system efficiency, environmental regulation stringency, or occupational safety standards—all of which are directly relevant to leukemia burden.

A constructive policy framing recognizes that China has achieved substantial progress (52.4% reduction in ASYLLR over 30 years) while identifying a clear improvement pathway: an additional 40% reduction would bring levels close to those in Japan. This gap is potentially modifiable through three intervention domains: (1) accelerating treatment protocol standardization and quality assurance mechanisms, (2) expanding hematology specialist capacity and telemedicine consultation networks to underserved regions, and (3) strengthening early detection targeting high-risk subgroups (males aged 20–29). Similarly, China’s 145.9% increase in age-standardized prevalence rate (ASPR) should be interpreted as reflecting treatment success—greater numbers of survivors requiring long-term care—necessitating healthcare system adaptation through the establishment of survivorship care infrastructure, comprehensive follow-up registries, and financial protection mechanisms for long-term care needs.

### 4.2 Unique evolutionary pattern of leukemia burden among Chinese young adults

The most striking finding of this study was the divergent trend of “rising morbidity burden with declining mortality burden” in leukemia among Chinese young adults. From 1990 to 2021, ASPR increased by 145.9%, age-standardized years lived with disability rate (ASYLDR) by 33.6%, while ASYLLR decreased by 52.4%. This pattern contrasts sharply with global trends—global ASPR increased by only 20.3% and ASYLDR decreased by 12.5% during the same period (3, 4).

Three mechanisms explain this morbidity-mortality paradox. First, advances in medical technology were the primary driver of mortality decline. China has made significant progress in leukemia treatment, with 5-year survival rates for childhood acute lymphoblastic leukemia (ALL) improving from less than 65% before 2014 to 90% afterward, approaching developed-country levels (14, 25). Standardization of hematopoietic stem cell transplantation and inclusion of targeted drugs (such as BTK inhibitors) in medical insurance have substantially improved patient prognosis (29, 30). Novel immunotherapies such as CAR-T cell therapy are also expanding in China (31, 32).

Second, the sustained increase in morbidity burden reflects the interplay of multiple complex factors. On one hand, improved survival rates have led to a growing number of surviving patients, positively reflecting medical progress. On the other hand, rising incidence cannot be ignored. This study found the fastest ASIR growth in the 20–29 age group (EAPC 0.69%), with male ASIR showing an upward trend (EAPC 0.307%), consistent with Li et al.’s report of 1.9% annual incidence growth from 2005 to 2017 (16). Environmental and lifestyle factors may serve as potential contributors or associated factors: with accelerating industrialization and urbanization in China, occupational exposure to benzene, ionizing radiation, and other chemical carcinogens has increased (13), combined with rising obesity rates (adult overweight rate 34.3%, obesity rate 16.4% during 2015–2019) (33), collectively elevating disease risk.

Third, the rise in ASYLDR warrants particular attention, especially the over 60% increase among males aged 20–29. This suggests that while treatment has extended survival, long-term health impairments from the disease and its treatment (such as organ dysfunction, secondary malignancies, and psychological issues) are becoming increasingly prominent (34). This phenomenon has also been reported among cancer survivors in other middle-income countries, highlighting the urgent need to transform care models from “saving lives” to “saving lives + improving quality of life.”

### 4.3 Underlying mechanisms of sex and age differences

This study found that male burden was significantly higher than female across all indicators, with widening sex gaps. In 2021, male ASIR (4.45/100,000) was 1.62 times that of females (2.74/100,000), with an even greater disparity in premature mortality burden (male 162.70 vs. female 101.19/100,000). From 1990 to 2021, male ASIR increased (EAPC 0.307%) while female ASIR decreased (EAPC -0.766%), further widening the sex gap.

This sex disparity is common in global leukemia research (2, 35), but the gap in China is more pronounced. Possible mechanisms include: biologically, X chromosome–carried immune-related genes confer stronger immune surveillance in females (36); behaviorally, Chinese male smoking rates (50.5%) far exceed those of females (2.1%) (37), with higher occupational benzene exposure risks; in terms of healthcare utilization, studies show males more commonly delay seeking care and exhibit poorer compliance.

Age distribution characteristics also warrant attention. This study found that the 20–24 age group bore the highest premature mortality burden (ASYLLR 151.21/100,000), while ASIR grew fastest in the 25–29 age group (EAPC 0.690%). This may relate to specific occupational exposures in this age range (entering the workforce and increased contact with industrial chemicals), lifestyle changes (high work pressure, irregular schedules and diet), and fertility-related factors (changes in female immune status during pregnancy). This finding echoes Huang et al.’s research on hematological tumor burden among young adults (7), suggesting that this age group should become a priority population for future screening and intervention.

### 4.4 Epidemiological transition of leukemia subtypes

This study revealed significant differences and evolutionary trends in burden across leukemia subtypes. ALL currently carries the heaviest burden, but CLL exhibits a “low incidence, high growth” pattern (ASIR EAPC 1.963%, ASYLDR EAPC 2.924%), heralding a potential future shift in the disease spectrum.

This trend shows both similarities and differences with global patterns. Global studies indicate that AML burden is heavier in high-SDI regions, while ALL dominates in low-SDI regions (38). The dominant position of ALL among Chinese young adults in this study is consistent with China’s transitional characteristics as a high-middle SDI country.

CLL’s rapid growth is particularly noteworthy, as this disease has historically had extremely low incidence in Asian populations, termed the “CLL mystery” (39). Despite having the lowest age-standardized incidence rate (ASIR 0.55/100,000), CLL accounted for the highest absolute disease burden in 2021, with 7,165 incident cases (32.6% of total leukemia incidence) and 61,396 prevalent cases (48.6% of total prevalence), both surpassing ALL. This apparent paradox of low age-standardized rates but high absolute burden primarily stems from CLL cases being concentrated in the older age groups within the 15–39 range (particularly ages 30–39, where population denominators are larger), combined with longer disease duration and survival. The substantial absolute case numbers (representing a 2.4-fold increase from an estimated 25,580 prevalent cases in 1990) indicate that the observed growth trend reflects meaningful population-level change rather than a statistical artifact from small baseline numbers. Rising CLL incidence in China may be related to improved diagnostic capabilities, population aging within the young adult cohort, and Westernization of environment and lifestyle (40), although the indolent nature of CLL suggests that enhanced detection of previously undiagnosed cases likely contributes to the observed trends.

Notably, CML showed the most significant declining trends (ASIR EAPC -2.093%, ASYLLR EAPC -5.188%), directly reflecting the revolutionary impact of tyrosine kinase inhibitor (TKI) treatment, such as imatinib (27). Since its introduction in 2002 and gradual inclusion in medical insurance, China has significantly improved CML patient prognosis, with 5-year survival rates rising from less than 30% to over 90% (28).

### 4.5 Future burden projection and policy implications

BAPC model projections showed that ASIR will moderately decline (from 3.556 to 3.335 per 100,000) while ASPR will substantially increase (from 19.38 to 33.08 per 100,000), a 70.6% rise, for Chinese young adult leukemia during 2023–2035. This projection is similar to Huang et al.’s predicted trends for global young adult hematological tumors (7), but China’s ASPR growth rate is faster.

These trends have important implications for health policy and resource allocation. First, sustained ASPR increase means the healthcare system will face growing long-term care demands. According to projections, approximately 200,000 leukemia patients aged 15–39 will be surviving in China by 2035, requiring continuous follow-up monitoring, complication management, and rehabilitation support (41). Second, rising ASYLDR (from 1.86 to 2.36 per 100,000) suggests the need to strengthen survivor health management, including late effects monitoring, psychological support, and vocational rehabilitation. Third, while continued ASYLLR decline (from 124.43 to 85.58 per 100,000) is positive, levels remain far higher than in developed countries, indicating substantial room for improvement in treatment accessibility and standardization.

However, projection uncertainty warrants careful consideration. Models extrapolate future trends from historical patterns and cannot capture the impact of disruptive factors such as policy interventions and technological breakthroughs. For example, if CAR-T therapy is widely scaled up or novel targeted drugs achieve breakthroughs, disease trajectories could change significantly. Confidence intervals widened after 2030, reflecting greater long-term projection uncertainty and underscoring the need for adaptive surveillance systems to respond to potential policy or technological changes. Therefore, projection results should serve as planning references rather than deterministic prophecies and require regular updates and corrections.

### 4.6 Innovation and insights from quality of care assessment

The QCI constructed in this study provides an innovative tool for evaluating the full chain of leukemia prevention, control, and care. Our analysis revealed a strong positive correlation between QCI and SDI (Spearman’s *ρ* = 0.77, *p* < 0.001), consistent with the expectation that higher socioeconomic development facilitates better healthcare quality (Figure 7). This pattern aligns with findings from all-age leukemia populations, which have demonstrated improved outcomes in higher-resource settings, although the strength of association observed here (ρ = 0.77) suggests particularly robust alignment between development level and care quality for the AYA population (42, 43).

China achieved a QCI of 74.3%, substantially exceeding the global average (55.2%) and ranking above most regions with comparable SDI (0.722). Multidimensional analysis revealed that China outperformed global averages across all five QCI components: care effectiveness index (75.5% vs. global 53.9%), burden control index (77.8% vs. 58.0%), survival quality index (67.7% vs. 44.7%), and recovery efficiency index (79.6% vs. 64.5%) (Figure 8).

These findings suggest that China’s leukemia care for AYAs has made substantial progress, likely attributable to multiple factors: standardization of treatment protocols through multi-institutional collaborative networks (14), expanded health insurance coverage for targeted therapies including tyrosine kinase inhibitors and novel immunotherapies (29–32), and improved access to hematopoietic stem cell transplantation (30).

However, despite the favorable overall QCI, China’s performance in specific dimensions still lags behind high-SDI regions. The survival quality index (67.7%) remained below the level observed in high-SDI regions (78.2%), indicating room for further improvement in long-term survivorship care. This gap may reflect challenges in comprehensive follow-up systems, late effects monitoring, and rehabilitation services for the growing population of young adult survivors (41). Similarly, while China’s recovery efficiency index (79.6%) exceeded the global average, it remained slightly below high-SDI levels (79.2%), suggesting opportunities to optimize long-term disease management.

The QCI framework, while useful, has important limitations. It relies on aggregate epidemiological ratios derived from population-level data and may not capture patient-centered outcomes such as quality of life, functional status, or psychosocial well-being (34). It cannot measure treatment delays, equity of access across geographic regions and socioeconomic strata, or adherence to evidence-based guidelines—all critical dimensions of care quality. The strong positive correlation with SDI, while reassuring, also raises questions about the specific mechanisms driving this association: is it healthcare system financing, medical technology availability, health literacy, or broader social determinants? Future refinements should incorporate process indicators, patient-reported outcomes, and subnational analyses to provide a more comprehensive assessment of care quality and to identify modifiable targets for intervention.

An intriguing juxtaposition emerges when comparing China’s high QCI score with its persistently elevated premature mortality (ASYLLR) relative to high-income countries. This apparent paradox may be resolved by considering that the QCI, as constructed here, likely reflects the quality of management for the existing pool of patients. In contrast, ASYLLR is more sensitive to “upstream” factors such as delays in diagnosis and inequitable access to first-line curative treatments across different regions and socioeconomic strata. Thus, while China has excelled in managing patients once they are within the healthcare system, the remaining burden of premature death highlights critical gaps in early detection and the universal reach of high-quality care.

### 4.7 Public health implications and policy recommendations

Based ased on the study findings, the following policy recommendations are proposed. First, establish a three-tier prevention system for young adult leukemia. Primary prevention should focus on risk factor control, including strengthening occupational health supervision, controlling benzene and other chemical exposures, and promoting tobacco control and obesity prevention. Targeted health education and risk screening should be conducted for the high-risk 20–29 age group. Secondary prevention should pilot early screening programs in high-risk populations, utilizing new technologies such as liquid biopsy to improve early diagnosis rates. Tertiary prevention should strengthen long-term follow-up management, establish care guidelines for young cancer survivors, and address psychological health, fertility preservation, and vocational rehabilitation.

Second, optimize medical resource allocation and service accessibility. Expand medical insurance coverage for targeted drugs and innovative therapies to reduce patient economic burden. Strengthen leukemia diagnosis and treatment capacity in primary healthcare facilities to narrow urban–rural and regional gaps (26). Promote tiered diagnosis and treatment and multidisciplinary collaboration to improve treatment standardization.

Third, improve social security systems for young cancer patients. Establish targeted economic assistance mechanisms, such as catastrophic disease insurance preferences, commercial insurance innovative products, and standardized social crowdfunding platforms (44). Strengthen employment protection and vocational rehabilitation support to help patients return to society.

Fourth, strengthen monitoring and research system construction. Establish a national young adult leukemia registry to improve data quality and completeness (45). Conduct etiological research to clarify China-specific risk factors. Strengthen international cooperation to learn from developed-country experiences.

### 4.8 Study limitations

First, the GBD database relies on multi-source data integration and statistical modeling, potentially introducing estimation bias, particularly in regions with poor data quality (46). Incomplete leukemia surveillance system coverage in some Chinese regions may lead to underestimation of disease burden. Additionally, for international comparisons, observed differences may partially reflect measurement artifacts—such as temporal changes in diagnostic sensitivity or data quality improvements—rather than true epidemiological disparities, making it difficult to definitively partition international gaps into “real” versus “artifactual” components.

Second, secondary data analysis cannot obtain individual-level detailed information such as specific risk factor exposures, treatment regimens, and clinical outcomes, limiting in-depth exploration of disease burden drivers. This constraint particularly affects our ability to establish causal mechanisms for observed international differences; while we hypothesize drivers such as healthcare system differences, environmental exposures, and genetic susceptibility, claims regarding policy-modifiable versus non-modifiable factors remain speculative without individual-level data.

Third, prediction models based on historical trends cannot capture the impact of future policy changes and technological breakthroughs, introducing prediction uncertainty. Fourth, while QCI construction was based on multiple indicators, it could not incorporate process indicators of treatment quality (such as treatment delays, regimen compliance) and patient-reported outcomes (such as quality of life), potentially failing to comprehensively reflect care quality.

Fifth, this study mainly focused on population-level burden at the macro level, without in-depth analysis of socioeconomic status, geographic region, ethnicity, and other subgroup differences, which are crucial for health equity research. Notably, aggregate national comparisons may obscure important subnational heterogeneity—China’s internal variation in leukemia outcomes between regions like Shanghai and western provinces likely exceeds variation between China’s national average and peer countries, suggesting that within-China spatial analyses may yield more actionable policy insights than additional international benchmarking. These limitations suggest that international comparisons presented in this study are most valuable for hypothesis generation and identifying potential improvement areas rather than for drawing definitive conclusions about relative health system performance.

Sixth, the Quality of Care Index (QCI) was constructed based on population-level epidemiological indicators rather than direct clinical care processes or patient-reported outcomes. The validity and reliability of QCI in reflecting real-world leukemia care quality require further verification.

### 4.9 Future research directions

Future research should deepen in the following directions. First, conduct population-based cohort studies to clarify specific risk factors for Chinese young adult leukemia, particularly the interaction of environmental exposures, lifestyle, and genetic susceptibility (47). Second, establish high-quality clinical registry databases to collect detailed treatment and follow-up information and evaluate care quality in actual clinical practice. Third, conduct health economic evaluations to quantify economic impacts of disease burden and provide cost-effectiveness evidence for resource allocation. Fourth, focus on health equity, exploring disparities in disease burden and care quality across socioeconomic status, urban–rural, and regional dimensions and their root causes. Fifth, utilize artificial intelligence and big data technologies to develop disease risk prediction models and personalized treatment decision support systems.

## 5 Conclusion

This study systematically assessed the current status, trends, and quality of care for leukemia burden among Chinese AYAs aged 15–39, filling an important knowledge gap in this field. The study found that Chinese young adult leukemia exhibits a unique “rising morbidity with declining mortality” pattern, with projected continued growth in future prevalence burden, and care quality showing significant progress but multiple shortcomings remaining. These findings have important implications for optimizing disease prevention and control strategies, improving care quality, and promoting health equity. At the national strategic level, recommendations include establishing comprehensive prevention and control systems, strengthening healthcare service accessibility, improving social security systems, and continuously conducting monitoring and research to inform more targeted provincial and local interventions, ultimately contributing to the “Healthy China 2030” Planning Outline goal of reducing premature mortality from major chronic diseases and safeguarding young people’s health.

## Data Availability

Publicly available datasets were analyzed in this study. This data can be found here: https://vizhub.healthdata.org/gbd-results/.
